# Tricuspid annular plane systolic excursion/mitral annular plane systolic excursion ratio in critically ill patients: an index of right- and left-ventricular function mismatch and a risk factor for cardiogenic pulmonary edema

**DOI:** 10.1186/s12871-023-02142-9

**Published:** 2023-05-22

**Authors:** Hongmin Zhang, Hui Lian, Xiaoting Wang, Qing Zhang, Dawei Liu

**Affiliations:** 1 Department of Critical Care Medicine, Peking Union Medical College Hospital, Chinese Academy of Medical Sciences, 1# Shuai Fu Yuan, Dong Cheng District, Beijing, 100730 China; 2 Department of Health Care, Peking Union Medical College Hospital, Chinese Academy of Medical Sciences and Peking Union Medical College, Beijing, China

**Keywords:** Pulmonary edema, Left ventricular dysfunction, Right ventricular dysfunction, Critically ill

## Abstract

**Background:**

This study aimed to explore whether the tricuspid annular systolic excursion (TAPSE)/mitral annular systolic excursion (MAPSE) ratio was associated with the occurrence of cardiogenic pulmonary edema (CPE) in critically ill patients.

**Materials and methods:**

This was a prospective observational study conducted in a tertiary hospital. Adult patients admitted to the intensive care unit who were on mechanical ventilation or in need of oxygen therapy were prospectively screened for enrolment. The diagnosis of CPE was determined based on lung ultrasound and echocardiography findings. TAPSE ≥ 17 mm and MAPSE ≥ 11 mm were used as normal references.

**Results:**

Among the 290 patients enrolled in this study, 86 had CPE. In the logistic regression analysis, the TASPE/MAPSE ratio was independently associated with the occurrence of CPE (odds ratio 4.855, 95% CI: 2.215–10.641, *p* < 0.001). The patients’ heart function could be categorized into four types: normal TAPSE in combination with normal MAPSE (TAPSE↑/MAPSE↑) (n = 157), abnormal TAPSE in combination with abnormal MAPSE (TAPSE↓/MAPSE↓) (n = 40), abnormal TAPSE in combination with normal MAPSE (TAPSE↓/MAPSE↑) (n = 50) and normal TAPSE in combination with abnormal MAPSE (TAPSE↑/MAPSE↓) (n = 43). The prevalence of CPE in patients with TAPSE↑/MAPSE↓ (86.0%) was significantly higher than that in patients with TAPSE↑/MAPSE↑ (15.3%), TAPSE↓/MAPSE↓ (37.5%), or TAPSE↓/MAPSE↑ (20.0%) (*p* < 0.001). The ROC analysis showed that the area under the curve for the TAPSE/MAPSE ratio was 0.761 (95% CI: 0.698–0.824, *p* < 0.001). A TAPSE/MAPSE ratio of 1.7 allowed the identification of patients at risk of CPE with a sensitivity of 62.8%, a specificity of 77.9%, a positive predictive value of 54.7% and a negative predictive value of 83.3%.

**Conclusions:**

The TAPSE/MAPSE ratio can be used to identify critically ill patients at higher risk of CPE.

**Supplementary Information:**

The online version contains supplementary material available at 10.1186/s12871-023-02142-9.

## Background

Acute cardiogenic pulmonary edema (CPE) is a common medical emergency and its prevalence continues to rise over time; an estimated 6.2 million American adults ≥ 20 years of age had heart failure between 2013 and 2016 [1]. CPE is among the major causes of acute respiratory failure and can result in higher mortality, longer hospital stay and increased cost [2–4]. Lung ultrasound and echocardiography can be used to detect CPE with the aid of clinical data [5]. The widespread use of echocardiography in the intensive care unit (ICU) also enables physicians to identify various types of myocardial dysfunction at bedside [6].

Left ventricular (LV) systolic dysfunction and/or LV diastolic dysfunction are usually deemed the main risk factors for acute respiratory failure or weaning failure from mechanical ventilation [7–10]. However, the mismatch between right ventricular (RV) and LV stroke volumes is a prerequisite for acute pulmonary edema to occur as fluid is lost from the circulation into the airspaces [11, 12]. It appears to be a basic concept that a strong RV might contribute to the occurrence of CPE in patients with LV dysfunction. However, which index might help assess RV and LV function mismatch and whether their mismatch was associated with CPE has seldomly been reported. Tricuspid annular plane systolic excursion (TAPSE) is a commonly used parameter of RV systolic function [13]. Similarly, mitral annular plane systolic excursion (MAPSE), a parameter that can be easily measured at the bedside, can reflect LV longitudinal systolic function as well as LV diastolic function [14, 15]. We hypothesize that the TAPSE/MAPSE ratio can be used as an index of RV-LV function mismatch, which constitutes a risk factor for CPE in critically ill patients. Thus, the aim of this study was to assess whether the TAPSE/MAPSE ratio is associated with the occurrence of CPE in critically ill patients.

## Methods

### Study population

This prospective observational study screened adult patients who were admitted to the ICU of a tertiary hospital from 1 May 2018 to 1 March 2021.

The inclusion criteria were as follows: patients on mechanical ventilation or those in need of oxygen therapy to maintain arterial SpO_2_ above 90%. Patients were excluded if they met any of the following criteria: age below 18 years; admitted after thoracotomy; history of chronic heart failure; moderate-to-severe chronic pulmonary hypertension; diffuse parenchymal lung disease; pneumothorax or subcutaneous emphysema; moderate-to-severe mitral or aortic valve disease; ACS complicated by ventricular septal rupture; insufficient image quality for echocardiography measurement; absence of an echocardiography examiner; or refusal to provide informed consent.

### Lung ultrasound and echocardiography

Lung ultrasound and echocardiograms were recorded within 24 h of ICU admission. One experienced physician (H Z) who was blinded to the patients’ clinical data performed the lung ultrasound and echocardiographic examination. Images were saved for offline analysis.

We scanned four chest areas per side to evaluate the presence of interstitial syndrome including the upper anterior, lower anterior, upper lateral and basal lateral areas with patients in the supine position [16]. The echocardiographic results were reported based on the PRICES statement [17]. At least three cardiac cycles were analysed and averaged. M-mode and Doppler echocardiographic measurements were taken according to standard protocols. The measurements of tricuspid annular plane systolic excursion (TAPSE), RV fractional area change (FAC), peak velocity of tricuspid regurgitation (TR), left ventricular ejection fraction (LVEF), mitral annular plane systolic excursion (MAPSE), mitral peak E velocity, averaged tissue Doppler velocity of lateral and medial mitral annuli at early diastole (e’), left ventricular outflow tract velocity-time integral (LVOT-VTI) and diameter of inferior vena cava (DIVC) were performed as previously described [18, 19]. Left atrial (LA) volume was measured based on tracings of the blood-tissue interface on apical four- and two-chamber views, which was then indexed to body surface area [20]. TAPSE ≥ 17 mm and MAPSE ≥ 11 mm were used as normal references [13, 21].

**Fig. 1:**
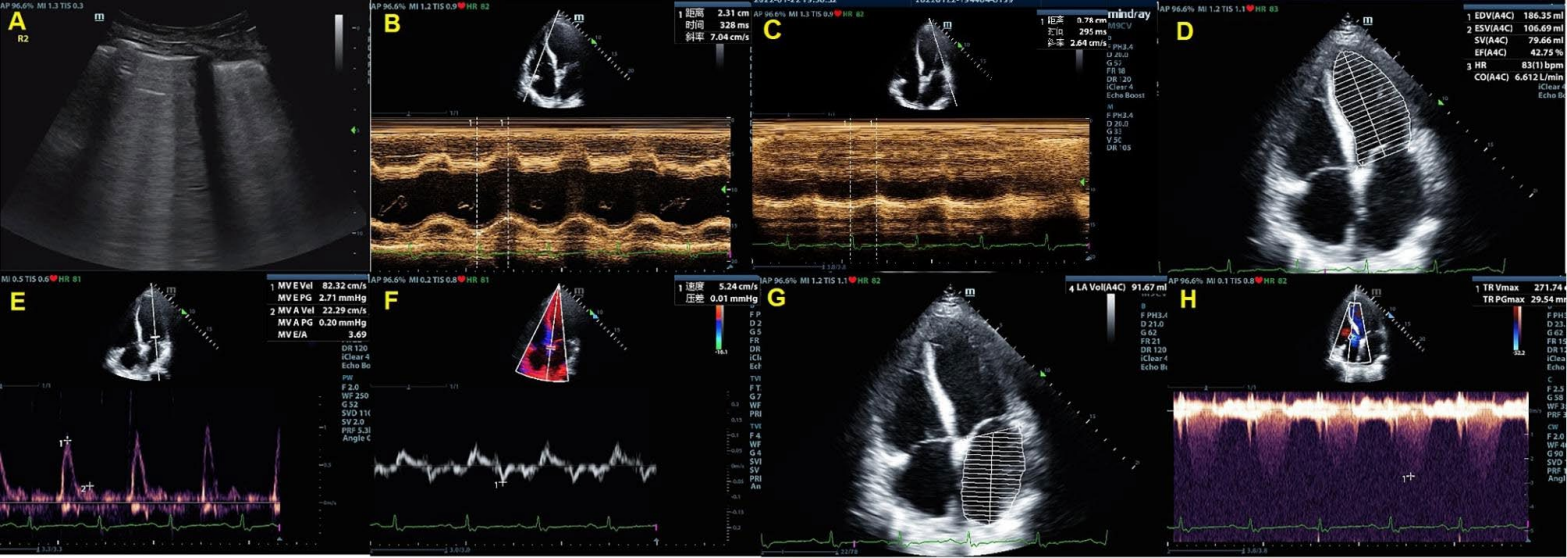
Lung ultrasound and echocardiographic examination. **A**. Lung ultrasound showing diffuse B-lines; **B**. TAPSE measurement; **C**. MAPSE measurement; **D**. LVEF measurement; **E**. Mitral E peak velocity; **F**. Septal e’ measurement; **G**. LA volume measurement; 1 H. TR measurement TAPSE: tricuspid annular plane systolic excursion; MAPSE: mitral annular plane systolic excursion; LVEF: left ventricular ejection fraction; LA: left atrium; TR: tricuspid regurgitation

CPE was diagnosed based on the integration of lung ultrasound and echocardiographic signs (Fig. 1): two or more regions with at least three B-lines bilaterally in the absence of pleural line abnormalities, reduction of lung sliding, anterior subpleural consolidations, and spared areas of normal parenchyma and E/e’ ≥ 13 or E/e’ 9–12 with at least 2 of the 4 specific conditions, namely, LAVI > 34ml/m^2^, PASP ≥ 40 mmHg, LVEF ≤ 45%, and LV hypertrophy [9, 22].

### Other parameters evaluated

Data on the following parameters for each patient were collected: heart rate (HR), mean arterial pressure (MAP), central venous pressure (CVP), mechanical ventilation (MV) support, norepinephrine (NE) use, and partial pressure of arterial oxygen to fraction of inspired oxygen ratio (PaO_2_/FiO_2_). Demographic information and data on the diagnosis, Acute Physiology and Chronic Health Evaluation (APACHE) II score, Sequential Organ Failure Assessment (SOFA) score, comorbidities, length of ICU stay and ICU mortality were also collected for all patients.

### Statistical analyses

Data analyses were performed using the statistical software package SPSS 22.0 (SPSS, Inc., Chicago, Illinois, USA). All *p* values were two tailed, and statistical significance was defined as *p* < 0.05. A previous study found a correlation between MAPSE and E/e’ in critically ill patients (r = 0.38) [14]. We anticipated at least a modest correlation between the TAPSE/MAPSE ratio and E/e’(r = 0.20–0.40). To detect an effect size of 0.20 at an alpha error of 0.05 and statistical power of 0.90, at least 259 participants were required for this study. All lung ultrasound and echocardiographic data were prospectively collected, and patients were excluded without TAPSE, MAPSE, LVEF, and E/e’ measurements due to inadequate echocardiographic image quality. Continuous data were expressed as the mean ± standard deviation or median (25th-75th percentiles). The distributions of the continuous values were assessed for normality by the Kolmogorov‒Smirnov test. Group differences were analysed using Student’s unpaired *t* test, the Mann‒Whitney U test, the chi-squared test, or Fisher’s exact test, as appropriate. We performed a binary logistic analysis to assess the independent factors of CPE. The variables that had *p* < 0.1 in the univariable model were included in the multivariable model and the odds ratio was calculated, together with their 95% confidence intervals (CI). Spearman’s correlation coefficients and their corresponding p values were calculated to assess the variable relationships. ROC curves were generated to determine the sensitivity and specificity of parameters to predict CPE. Sensitivity analyses were performed to test the TAPSE/MAPSE ratio for the detection of CPE in patients with normal LVEF and abnormal LVEF. Intra-observer variability in TAPSE, MAPSE and LVEF was assessed in 20 randomly selected patients and was tested using intraclass correlation coefficients (ICCs). An ICC > 0.8 was considered excellent agreement.

## Results

### Baseline characteristics of the study population

**Fig. 2:**
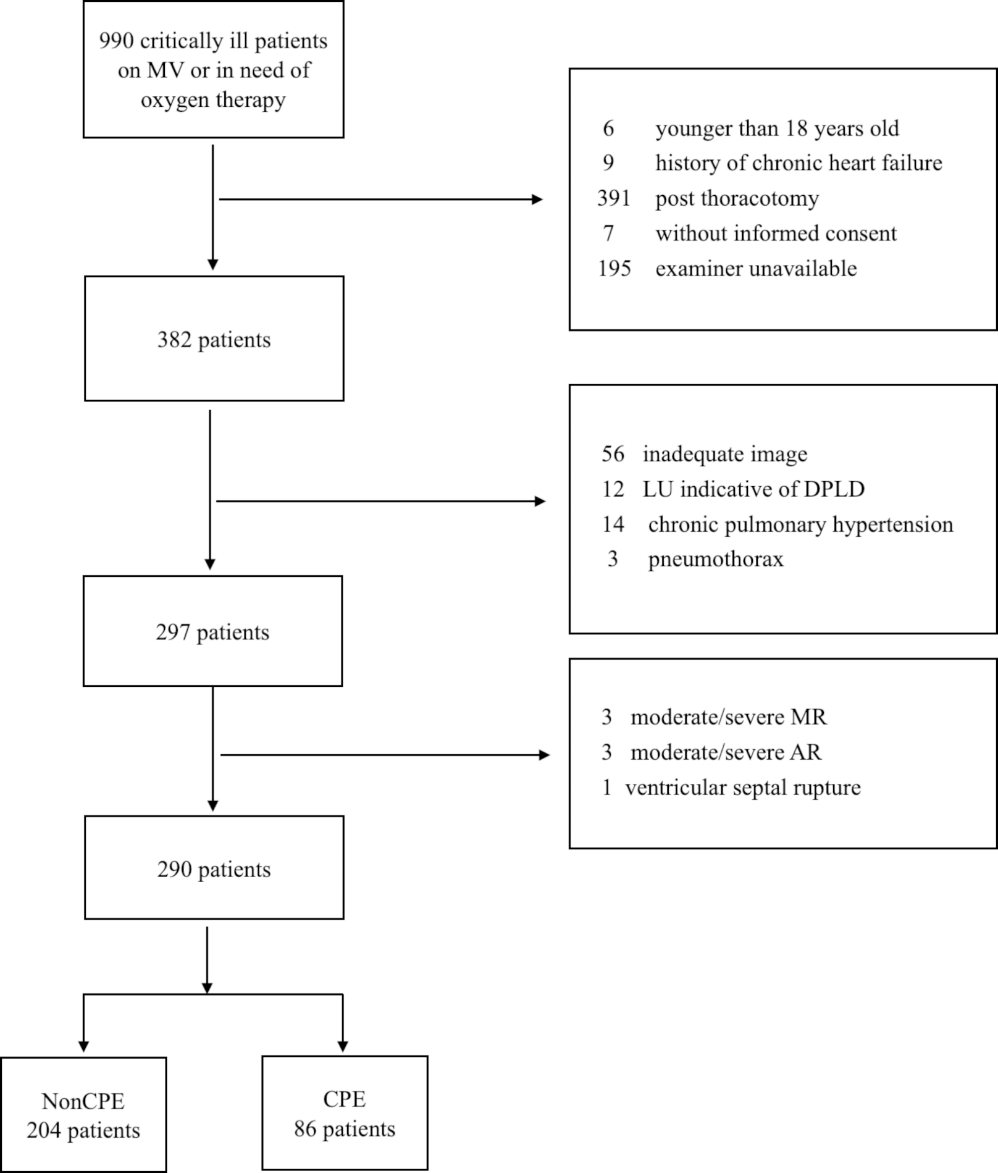
Flow chart of the study ICU: intensive care unit; DPLD: diffuse parenchymal lung disease; ARDS: acute respiratory distress syndrome; LU: lung ultrasound; MR: mitral regurgitation; AR: aortic regurgitation; CPE: cardiogenic pulmonary edema

During the study period, 990 critically ill patients on mechanical ventilation or in need of oxygen therapy were screened for enrolment, and 700 patients were excluded (Fig. 2). Among the 290 patients enrolled in this study, 86 were categorized into the CPE group, and the remaining 204 were categorized into the nonCPE group. Table 1 summarizes the general characteristics of the two groups. No significant differences were found between the two groups regarding sex, illness severity, comorbidities, or length of ICU stay. The two groups had similar proportions of sepsis and MV support. In comparison with the nonCPE group, the CPE group had older age (66 vs. 63, *p* = 0.007), lower PaO_2_/FiO_2_ (236 vs. 323 mmHg, *p* < 0.001) and higher ICU mortality (22.1% vs. 11.3%, *p* = 0.017).

**Table 1:** Baseline characteristics of the study population

Categories	Study population (n = 290)	NonCPE (n = 204)	CPE (n = 86)	*p* value
Age (yr)	64 (52, 73)	63 (50, 72)	66 (58, 79)	0.007
Sex (male, %)	184 (62.2%)	126 (61.8%)	58 (63.0%)	0.834
APACHE II	19 (13, 24)	18 (14, 24)	20 (13, 25)	0.399
SOFA	10 (8, 13)	10 (8, 13)	11 (6, 13)	0.639
Reasons for admission (n, %)				
Sepsis	172 (59.3%)	123 (60.3%)	49 (57.0%)	0.599
High-risk surgery	58 (20.0%)	44 (21.6%)	14 (16.3%)	0.304
*Others	60 (20.7%)	37 (18.1%)	23 (26.7%)	0.098
Comorbidities				
HTN	107 (36.9%)	72 (35.3%)	35 (40.7%)	0.384
DM	70 (24.1%)	45 (22.1%)	25 (29.1%)	0.203
CAD	59 (20.3%)	36 (17.6%)	23 (26.7%)	0.079
CKD	24 (8.3%)	14 (6.8%)	10 (11.6%)	0.179
COPD	13 (4.5%)	10 (4.9%)	3 (3.5%)	0.595
NE infusion (n, %)	211 (72.8%)	151 (74.0%)	60 (69.8%)	0.458
Mechanical ventilation (n, %)	241 (83.1%)	173 (84.8%)	68 (79.1%)	0.234
PaO_2_/FiO_2_	306 (247, 348)	323 (272, 359)	236 (191, 308)	< 0.001
ICU length of stay (day)	5 (3, 11)	5 (3, 10)	6 (3, 14)	0.096
ICU mortality (n, %)	42 (14.5%)	23 (11.3%)	19 (22.1%)	0.017

*Brain trauma or hemorrhage, acute kidney failure, acute coronary syndrome, sepsis without shock

CPE: cardiogenic pulmonary edema; APACHE: acute physiology and chronic health evaluation; SOFA: sequential organ failure assessment; HTN: hypertension; DM: diabetes mellitus; CAD: coronary arterial disease; CKD: chronic kidney dysfunction; COPD: chronic obstructive pulmonary disease; NE: norepinephrine; PaO_2_/FiO_2_: partial pressure of arterial oxygen to fraction of inspired oxygen ratio; ICU: intensive care unit

### Comparison of hemodynamic and echocardiographic parameters between the two groups

The lung ultrasound, TAPSE, FAC, MAPSE, E/e’, LAVI, and LVEF data were complete. TR was undetectable in 27 patients, IVCD was unavailable in 15 patients and LVOT-VTI was unavailable in 6 patients. The two groups had similar HRs and MAPs. The CPE group had a higher CVP level than the nonCPE group (*p* = 0.008). No significant difference was found regarding TAPSE (*p* = 0.657) and mitral peak A wave velocity (*p* = 0.509) between the two groups. The CPE group had lower FAC, LVEF, e’ velocity and cardiac index (CI) than the nonCPE group (*p* < 0.05). The CPE group had higher mitral peak E wave velocity, E/e’ ratio, LAVI, TR and DIVC than the nonCPE group (*p* < 0.001) (Table 2).

**Table 2:** Hemodynamic and echocardiographic parameters of the two groups

	Study population (n = 290)	NonCPE (n = 204)	CPE (n = 86)	*p* Value
HR (bpm)	93 ± 18	94 ± 18	93 ± 18	0.506
MAP (mmHg)	81 (72, 88)	81 (72, 87)	82 (73, 91)	0.219
CVP (mmHg)	8 (6, 10)	8 (6, 10)	9 (7, 12)	0.008
TAPSE (mm)	19.0 ± 5.1	19.1 ± 5.2	18.8 ± 5.0	0.657
FAC (%)	44 ± 12	46 ± 12	41 ± 13	0.001
LVEF (%)	58 (43, 68)	63 (51, 69)	43 (35, 55)	< 0.001
MAPSE	12.6 ± 3.8	13.7 ± 3.5	10.1 ± 3.1	< 0.001
TAPSE/MAPSE ratio	1.5 (1.3, 1.9)	1.4 (1.2, 1.7)	1.9 (1.4, 2.4)	< 0.001
E velocity (cm/s)	70 (56, 88)	63 (52, 76)	97 (85, 110)	< 0.001
A velocity (cm/s)	73 (57, 87)	73 (59, 85)	67 (42, 99)	0.509
e’(cm/s)	7.8 (6.2, 9.4)	8.3 (6.7, 10.0)	6.5 (4.9, 7.5)	< 0.001
E/e’	8.7 (6.6, 11.9)	7.6 (6.2, 9.7)	15.1 (12.7, 18.5)	< 0.001
LAVI (ml/m^2^)	35 (26, 46)	32 (24, 46)	42 (32, 50)	< 0.001
TR(m/s)	2.3 (2.1, 2.6)	2.2 (2.0, 2.5)	2.6 (2.4, 3.0)	< 0.001
PASP	27.5 (22.5, 35.6)	26.1 (21.2, 32.8)	35.0 (27.7, 45.9)	< 0.001
DIVC (mm)	13 ± 4.4	16.3 ± 4.4	19.9 ± 4.3	< 0.001
LVOT-VTI (cm)	17.2 ± 4.6	17.4 ± 4.5	16.2 ± 5.0	0.087
CI (L/min/m^2^)	3.3 (2.6, 3.9)	3.3 (2.7, 3.9)	3.1 (2.4, 3.6)	0.033

CPE: cardiogenic pulmonary edema; HR: heart rate; MAP: mean arterial pressure; CVP: central venous pressure; TAPSE: tricuspid annular plane systolic excursion; MAPSE: mitral annular plane systolic excursion; FAC: right ventricular fractional area change; TR: tricuspid regurgitation; LVEF: left ventricular ejection fraction; e’: mitral e’ velocity; DIVC: diameter of inferior vena cava; LVOT-VTI: left ventricular outflow tract velocity-time integral; CI: cardiac index

### Factors associated with the presence of CPE

In the logistic regression analysis, age (OR 1.028, 95% CI:1.005–1.052, *p* = 0.016), CVP (OR 1.148, 95% CI: 1.021–1.291, *p* = 0.022), DIVC (OR 3.995, 95% CI:1.554–10.274, *p* = 0.004), LVEF (OR 0.953, 95% CI: 0.928–0.978, *p* < 0.001), and the TAPSE/MAPSE ratio (OR 4.855, 95% CI: 2.215–10.641, *p* < 0.001) were independently associated with the occurrence of CPE (Table 3).

**Table 3:** Factors associated with the occurrence of CPE

	Odds Ratio	95%CI	*p* Value
Univariable analysis			
Age	1.022	1.006–1.039	0.007
CVP	1.143	1.051–1.242	0.002
DIVC	5.765	3.010-11.041	< 0.001
LVEF	0.936	0.918–0.955	< 0.001
FAC	0.964	0.944–0.986	0.001
CI	0.722	0.540–0.965	0.028
TAPSE/MAPSE ratio	6.779	3.812–12.053	< 0.001
Multivariable analysis			
Age	1.028	1.005–1.052	0.016
CVP	1.148	1.021–1.291	0.022
DIVC	3.995	1.554–10.274	0.004
LVEF	0.953	0.928–0.978	< 0.001
TAPSE/MAPSE ratio	4.855	2.215–10.641	< 0.001

CPE: cardiogenic pulmonary edema; CVP: central venous pressure; DIVC: diameter of inferior vena cava; LVEF: left ventricular ejection fraction; TAPSE: tricuspid annular plane systolic excursion; MAPSE: mitral annular plane systolic excursion

### Prevalence of CPE in patients with different ventricular functions

**Fig. 3:**
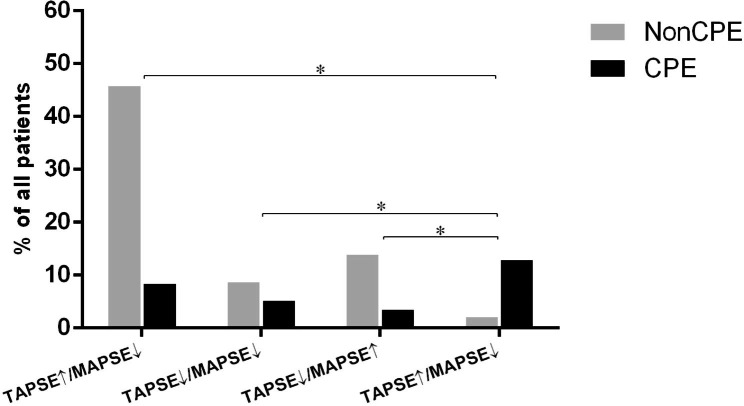
Prevalence of CPE in patients with different ventricular functions. The prevalence of CPE in patients with patients with TAPSE↑/MAPSE↓ (86.0%) was significantly higher than patients with TAPSE↑/MAPSE↑ (15.3%), TAPSE↓/MAPSE↓ (37.5%) and TAPSE↓/MAPSE↑ (20.0%) (*p* < 0.001) CPE: cardiogenic pulmonary edema; TAPSE: tricuspid annular plane systolic excursion; MAPSE: mitral annular plane systolic excursion; TAPSE↑/MAPSE↑: normal TAPSE in combination with normal MAPSE; TAPSE↓/MAPSE↓: abnormal TAPSE in combination of abnormal MAPSE; TAPSE↓/MAPSE↑: abnormal TAPSE in combination with normal MAPSE; TAPSE↑/MAPSE↓: normal TAPSE in combination with abnormal MAPSE.

Among all the patients, 83 (28.6%) displayed abnormal MAPSE and 90(31.0%) displayed abnormal TAPSE. We categorized the patients’ ventricular function into four types: normal TAPSE in combination with normal MAPSE (TAPSE↑/MAPSE↑) (n = 157), abnormal TAPSE in combination with abnormal MAPSE (TAPSE↓/MAPSE↓) (n = 40), abnormal TAPSE in combination with normal MAPSE (TAPSE↓/MAPSE↑) (n = 50) and normal TAPSE in combination with abnormal MAPSE (TAPSE↑/MAPSE↓) (n = 43). The prevalence of CPE in patients with TAPSE↑/MAPSE↓ (86.0%) was significantly higher than that in patients with TAPSE↑/MAPSE↑ (15.3%), TAPSE↓/MAPSE↓ (37.5%) or TAPSE↓/MAPSE↑ (20.0%) (*p* < 0.001) (Fig. 3).

### Relationship between the TAPSE/MAPSE ratio and CPE

**Fig. 4:**
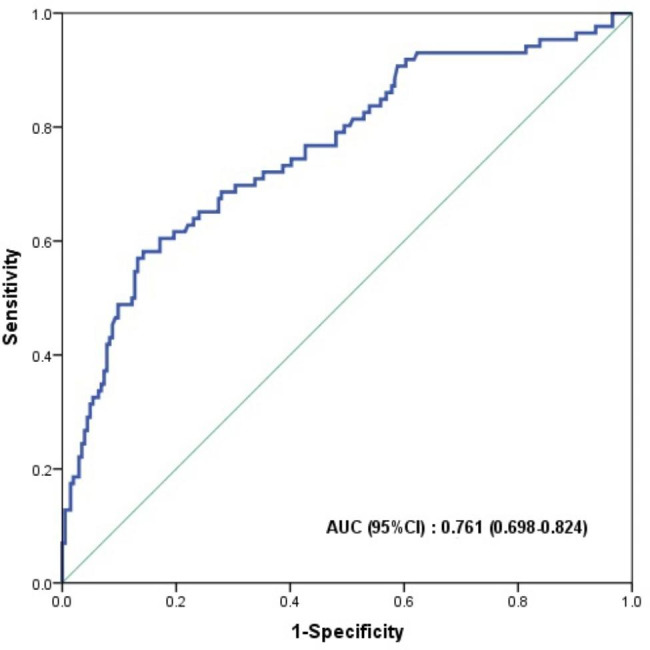
ROC analysis of the TAPSE/MAPSE ratio for the prediction of CPE. The ROC analysis showed that the areas under the curve for the TAPSE/MAPSE ratio for prediction of CPE was 0.761 (95%CI 0.698–0.824, *p* < 0.001) CPE: cardiogenic pulmonary edema; TAPSE: tricuspid annular plane systolic excursion; MAPSE: mitral annular plane systolic excursion

The TAPSE/MAPSE ratio was correlated with E/e’ (r = 0.343, *p* < 0.001). To evaluate the sensitivity and specificity of the TAPSE/MAPSE ratio in the prediction of CPE, ROC curves were generated. The ROC analysis showed that the area under the curve for the TAPSE/MAPSE ratio was 0.761 (95% CI: 0.698–0.824, *p* < 0.001). The optimum cut-off value of the TAPSE/MAPSE ratio for the prediction of CPE was 1.7, which resulted in a sensitivity of 62.8%, a specificity of 77.9%, a positive predictive value of 54.7% and a negative predictive value of 83.3% (Fig. 4).

### Sensitivity analysis

We performed sensitivity analysis in patients with LVEF ≥ 50% (n = 185) and in patients with LVEF < 50% (n = 105) separately and found that the AUCs of the TAPSE/MAPSE ratio for the detection of CPE were 0.679 (95% CI: 0.569–0.789, *p* = 0.003) and 0.743 (95% CI: 0.649–0.837, *p* < 0.001), respectively. We also performed subgroup analysis of hemodynamic and echocardiographic parameters among CPE patients with different types of ventricular function and found that patients with TAPSE↑/MAPSE↓ had similar E/e’ values and had significantly lower CVP than the other three types (*p* < 0.05) (Supplemental Fig. 1).

### Reproducibility

The intra-observer variability analysis revealed that the ICCs for TAPSE, MAPSE and LVEF were: 0.975 (95% CI: 0.937–0.990), 0.955 (95% CI: 0.890–0.982) and 0.930 (95% CI: 0.832–0.972), respectively.

## Discussion

In this study, we prospectively assessed the lung ultrasound and echocardiography of critically ill patients on mechanical ventilation or in need of oxygen therapy. We found that the TAPSE/MAPSE ratio was independently associated with the occurrence of CPE. We also found that patients with TAPSE↑/MAPSE↓ were common and were more prone to CPE than patients with TAPSE↑/MAPSE↑, TAPSE↓/MAPSE↓ or TAPSE↓/MAPSE↑.

We integrated lung ultrasound and echocardiography to detect CPE, which could be missed by clinical examination [23]. In comparison with a prior study on weaning failure patients, the PaO_2_/FiO_2_ ratio was higher in this study (median 306 vs. 167 mmHg) possibly due to the identification of CPE patients without evident respiratory stress [24]. Therefore, ultrasound examination could enable physicians to identify and manage CPE patients at an earlier stage, thus avoiding dire consequences.

This study revealed that normal RV function in combination with abnormal LV function was not rare in critically ill patients. In patients with acute coronary syndrome (ACS) or Takotsubo cardiomyopathy, isolated LV systolic dysfunction is common [25–27]. Hania et al. reported that within 5 days of myocardial infarction, LV systolic dysfunction was detected in 46% of AMI patients [28]. In a medical ICU, left ventricular apical ballooning was found in 28% of critically ill patients [27]. LV systolic function, LV diastolic function and RV function can be compromised independently or collectively during sepsis [29, 30]. Therefore, RV and LV function mismatch can also exist in septic patients.

We found that patients with normal TAPSE and lower MAPSE were prone to CPE. There is a close interrelationship between systolic and diastolic function. Energy is stored during systole, which will be released during early diastole improving forwards flow into the ventricle. If LV contractile function is reduced, diastolic forwards flow will be compromised [12]. Thus, diastolic function is usually worse in heart failure patients with reduced LVEF than in those with preserved LVEF [31]. Additionally, some researchers found that MAPSE had a close relationship with LV diastolic function in obese patients [15]. This study found that patients with TAPSE↑/MAPSE↓ had a higher prevalence of CPE than those with TAPSE↓/MAPSE↓ (86% vs. 37.5%), which suggests that RV systolic function contributes to the occurrence of CPE. A previous study on patients with acute heart failure also noted that preserved RV function was associated with the development of pulmonary edema [32]. Interestingly, Kobayashi et al. contended that impaired RV function was associated with severe pulmonary congestion [33]. However, they incorporated only patients with pulmonary congestion and focused on the severity rather than the risk factors for pulmonary edema. Furthermore, they enrolled only patients with decompensated heart failure. The pulmonary arterial pressure was much higher than that in this study, which suggested the existence of a longstanding higher RV afterload induced by LV failure.

This study reminds us to pay more attention to RV systolic function when dealing with CPE. For Patients with TAPSE↑/MAPSE↓, CPE could occur when CVP or DIVC was still lie in the “normal” range. A prior study on heart failure patients with reduced LVEF reported that β blockers were associated with lower mortality in patients with preserved RVEF but not in those with reduced RVEF [34]. This study also suggests that downregulating hyperdynamic RV could be an alternative treatment in critically ill patients with CPE. Adequate sedation or analgesia or administration of β blockers with close monitoring of cardiac output has the potential to reduce the adrenal level and thus improve the mismatch of RV and LV function. Therefore, this study provided us with a new perspective to address critically ill patients with CPE.

### Limitations

First, this is a single-centre observational study, and sepsis patients accounted for a large part of the enrolled population, which might affect the external validity. Furthermore, we could not identify the exact aetiologies for RV-LV function mismatch, which can result from hypertension, ACS, Takotsubo cardiomyopathy, septic cardiomyopathy or other causes. Second, lung ultrasound and echocardiography were not performed serially. Thus, we did not know how myocardial function and CPE developed. Future serial studies are warranted. Third, the differentiation between ARDS and CPE can be challenging [35]. However, the lung ultrasound and echocardiography examination enabled us to identify ARDS patients according to the specific pleural abnormalities and the lack of evidence of LV filling pressure elevation [36].

## Conclusion

The TAPSE/MAPSE ratio can be used to identify critically ill patients at higher risk of CPE.

## Electronic supplementary material

Below is the link to the electronic supplementary material.

Supplementary Material 1: Echocardiographic and hemodynamic parameters of CPE patients with different ventricular function.

## Data Availability

Data cannot be made available for the public because the patient consent did not include such an agreement. However, data may be made available for selected research questions on request to the corresponding author.

## Abbreviations

APACHE: Acute physiology and chronic health evaluation.
ARDS: Acute respiratory distress syndrome
CPE: Cardiogenic pulmonary edema
CVP: Central venous pressure
DIVC: Diameter of inferior vena cava
HR: Heart rate
ICU: Intensive care unit
LV: Left ventricle
LVEF: Left ventricular ejection fraction
MAPSE: Mitral annular plane systolic excursion FAC:fractional area change
NE: Norepinephrine
PEEP: Positive end-expiratory pressure
Pplat: Plateau pressure
RV: Right ventricle
SOFA: Sequential organ failure assessment
TAPSE: Tricuspid annular plane systolic excursion
