# Supplementary material for: Tricuspid annular plane systolic excursion/mitral annular plane systolic excursion ratio in critically ill patients: an index of right- and left-ventricular function mismatch and a risk factor for cardiogenic pulmonary edema
Source: BMC Anesthesiol. 2023 May 22;23:175. doi: 10.1186/s12871-023-02142-9 (PMC10201736; doi:10.1186/s12871-023-02142-9)
Supplement: Supplementary file 1 — Supplementary Material 1: Echocardiographic and hemodynamic parameters of CPE patients with different ventricular function. [file 12871_2023_2142_MOESM1_ESM.docx]

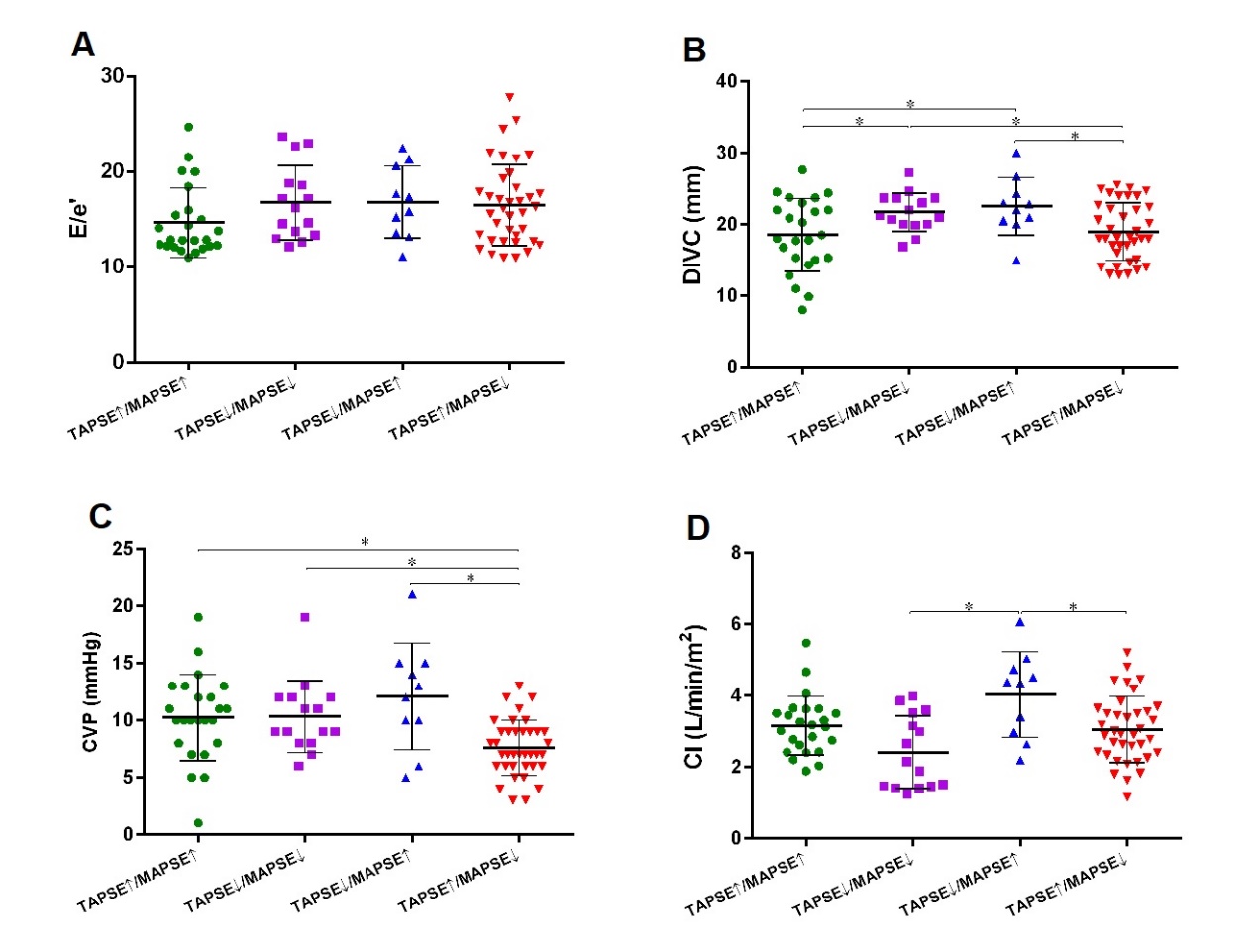


Supplemental Figure 1. Echocardiographic and hemodynamic parameters of CPE patients with different ventricular function.

Figure 1A. No difference was found on E/e’ among the four groups.

Figure 1B. The DIVC in patients with TAPSE↑/MAPSE↓was lower than patients with TAPSE↓/MAPSE↓ and TAPSE↓/MAPSE↑ (*p* <0.005).

Figure 1C. The CVP in patients with TAPSE↑/MAPSE↓ was lower than patients with TAPSE↓/MAPSE↓ and TAPSE↓/MAPSE↑ (*p* <0.005).

Figure 1D. The CI in patients with TAPSE↓/MAPSE↑ was higher than patients with TAPSE↓/MAPSE↓ and TAPSE↑/MAPSE↓ (*p* <0.005).
